# Eccentric-only versus concentric-only resistance training effects on biochemical and physiological parameters in patients with type 2 diabetes

**DOI:** 10.1186/s13102-021-00384-z

**Published:** 2021-12-20

**Authors:** Christine Kudiarasu, Wafina Rohadhia, Yoshihiro Katsura, Tomoko Koeda, Favil Singh, Kazunori Nosaka

**Affiliations:** 1 School of Medical and Health Sciences, Edith Cowan University, Joondalup, WA Australia; 2 Exercise Medicine Research Institute, Edith Cowan University, Joondalup, WA Australia; 3 Centre for Promotion of Higher Education, Kogakuin University, Tokyo, Japan; 4 Faculty of Rehabilitation Sciences, Nagoya Gakuin University, Aichi, Japan

**Keywords:** Insulin sensitivity, Lipid profile, Strength, Physical function, Body composition, Rate of perceived exertion

## Abstract

**Background:**

The benefits of resistance training for patients with type 2 diabetes (T2D) are well documented; however, the effects of exercise with different muscle contraction types such as eccentric versus concentric contractions on physiological outcomes for this population are not clear. This study compared eccentric-only (ECC) and concentric-only resistance training (CON) to test the hypothesis that ECC would be superior to CON to improve insulin sensitivity, lipid profile, body composition, muscle strength and physical function of patients with T2D.

**Methods:**

Adults with T2D (50–79 years) were allocated to the ECC (*n* = 9) or CON group (*n* = 9). Resistance exercises (chest press, lateral pulldown, bicep curl, triceps extension, leg extension, leg curl, calf raise, abdominal crunch) consisting of 2–3 sets of 10 eccentric-only (5 s) or concentric-only contractions (1–2 s) was performed twice a week for 12 weeks. Changes in blood biomarkers, body composition, muscle strength and physical function from pre- to post-intervention were compared between groups.

**Results:**

Overall rating of perceived exertion (RPE, 1–10 Borg scale) was lower (*p* < 0.05) for ECC (2.9 ± 1.2) than CON (5.4 ± 1.1). No significant changes in blood biomarkers were found for both groups. Lean mass increased [effect size (ES) = 0.148, ECC 3.2 ± 6.9%; CON 3.6 ± 2.3%], and fat mass decreased (ES = 0.545, ECC − 6.1 ± 12.4%; CON − 7.1 ± 16.4%) (*p* < 0.05) similarly. One-repetition maximal strength of each exercise increased (*p* < 0.05) for both ECC (12–37%) and CON (27–68%). Both groups improved (*p* < 0.05) 6-min walk distance (ES = 0.083, ECC 12.2 ± 2.3%; CON 12.5 ± 15.3%) and chair rise time (ES = 0.463, ECC − 13.4 ± 25.4%; CON − 20.0 ± 53.3%) but only ECC improved (*p* < 0.05) the timed up-and-go test (− 11.3 ± 13.6%, ES 0.014). No significant changes in balance tests were found for both groups.

**Conclusion:**

These results did not fully support the hypothesis but showed that ECC was as effective as CON to improve body composition, muscle strength, and physical function with lesser RPE. Future studies should investigate whether larger differences between ECC and CON are evident when increasing the exercise frequency and matching the intensities of the two-exercise protocols.

*Trial registration* ACTRN12621001026819 (retrospectively registered on 5th Aug 2021).

## Introduction

Type 2 diabetes (T2D) is one of the fastest growing chronic diseases and is a major burden on the global economy and healthcare system [8]. The International Diabetes Federation [20] predicted that more than half a billion people will have T2D by 2030. It is well documented that exercise is essential in managing T2D, with evidence showing that performing structured aerobic and resistance exercise for more than 150 min per week can improve insulin sensitivity and glycemic control [7]. However, T2D is often associated with other comorbidities which may limit the individual’s aerobic capacity, physical ability, and strength [32, 41] to perform physical activity. This may reduce exercise tolerance and adherence for patients with T2D to conventional exercise training [25]. Thus, developing an exercise intervention that is efficient and well tolerated for patients with T2D is vital.

Our daily activities consist of static (isometric), shortening (concentric) and lengthening (eccentric) muscle contractions (actions). Activities such as descending stairs, walking or running downhill, and lowering weights predominantly demand eccentric contractions in which contracting muscles are lengthened while resisting against load [27]. Muscles can generate more force maximally and can be loaded greater during eccentric than concentric contractions [17, 35]. Moreover, the metabolic demand of eccentric exercise is much less (25–50%) than concentric exercise at the same workload [24]. Hence, eccentric exercises (e.g., descending stair walking) can be performed with reduced perceived effort when compared with concentric exercises (e.g., ascending stair walking) [25]. These characteristics make eccentric exercises advantageous and beneficial for less fit individuals such as patients with chronic diseases [27].

Importantly, several studies have shown that eccentric exercise training is more effective than concentric exercise training in improving insulin sensitivity, glycemic control, blood lipid profile, and physical fitness in healthy individuals [4, 5, 10]. For example, Chen et al. [4] reported that progressive eccentric resistance training of the knee extensors performed once a week for 12 weeks improved insulin sensitivity, lipid profile, physical functional performance, and muscular strength greater than concentric resistance training in healthy elderly men. Drexel et al. [10] showed that walking downhill for eight weeks 3–5 times a week significantly improved insulin action, glucose and lipid metabolism and body mass index. Similarly, a recent study demonstrated that descending stair walking performed three times a week for 12 weeks significantly improved body composition and insulin sensitivity in young women who are obese [5]. Marcus et al. [30] investigated a combination of aerobic and eccentric resistance training (AE/ECC) against aerobic only training (AE) performed three times a week for 16 weeks in patients with T2D. They found greater improvements in HbA1c levels for the AE/ECC (− 0.59%) than AE (− 0.31%), and greater increases in thigh lean mass for the AE/ECC (15%) than AE group (− 6%). Additionally, the six-minute walk test (6MWT) increased by 48% and body mass index (BMI) decreased by 2% for the AE/ECC group only. These studies suggest that eccentric exercise may be beneficial not only in improving insulin sensitivity and glycemic control, but also improving physical function for people with T2D.

Previous eccentric exercise studies only used resistance exercise of an isolated single muscle group or aerobic mode of exercise as an intervention for patients with T2D [13]. To the best of our knowledge, no studies have investigated the effects of eccentric-only resistance training on insulin sensitivity, lipid profile, body composition, muscle strength and physical function in adults with T2D in comparison to concentric-only resistance training. Therefore, the primary aim of this study was to compare 12-weeks of whole-body progressive eccentric-only and concentric-only resistance training to examine its effects on insulin sensitivity, glycemic control and blood lipid profile in adults with T2D. Secondary outcome measures included body composition, muscle strength, and physical function. Based on the results of a previous study [4], it was hypothesised that eccentric-only resistance training would be more effective than concentric-only resistance training in improving all outcome measures.

## Methods

### Participants

**Fig. 1:**
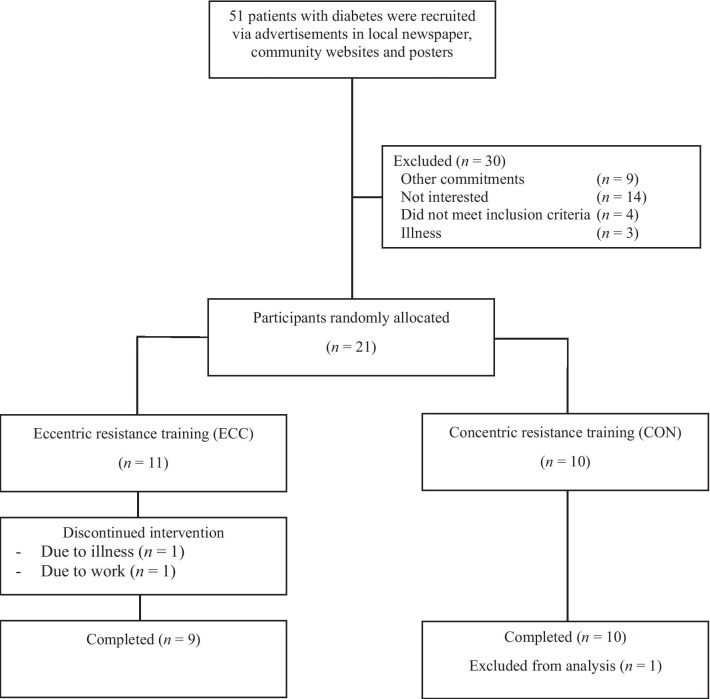
Consort diagram of participant recruitment

Fifty one adults who were diagnosed with T2D were screened, and 21 eligible participants were identified for this study (Fig. 1). The inclusion criteria were as follows: (1) adults aged 50–80 years, (2) fasting glucose > 7.0 mmol/L at the time of diagnosis, (3) no acute illness or any musculoskeletal, cardiovascular or neurological disorder that could inhibit exercise performance or put participants at risk from exercising, (4) no resistance training for at least 3 months prior to participation in the present study, and (5) medical clearance for participation. The participants provided written informed consent, and medical clearance gained from their general practitioner prior to participation in the study. They were instructed not to perform any resistance exercise or change their dietary habits during the experimental period. The study was approved by the Edith Cowan University Human Research Ethics Committee and was conducted in accordance with the Declaration of Helsinki.

Twenty one participants were stratified by age, gender, fasting glucose and previous physical activity level, and randomly allocated to either an ECC (*n* = 11) or a CON resistance training group (*n* = 10) as shown in Fig. 1. Two participants in the ECC group withdrew from the study due to illness or work commitment, and one participant in the CON group was excluded from the analysis, as this participant had much higher blood diabetes and lipid profile markers at baseline when compared with the rest of the participants. Thus, the final number of participants was nine for the ECC group [6 male, 3 female; mean ± SD (range) age 65.1 ± 9.8 (50–74) years, height 1.73 ± 0.12 (1.57–1.91) m, body mass 90.4 ± 16.3 (62.0–110.0) kg, and BMI 30.3 ± 5.2 (21.9–36.7) kg/m^2^], and also nine for the CON group [7 male, 2 female; age: 63.2 ± 8.6 (50–79) years, height: 1.74 ± 0.10 (1.58–1.87) m, body mass: 89.5 ± 15.3 (75.1–108.3) kg, and BMI: 29.4 ± 3.8 (24.8–33.7) kg/m^2^]. No significant differences between groups were found for the baseline measures.

### Study design

The exercise intervention period was 12 weeks, and testing was conducted at baseline (week 0), mid-intervention (week 6) and post-intervention (week 13). Prior to the commencement of the exercise intervention, the participants attended two separate sessions: (1) the first session to obtain baseline measurements (fasting blood test, body composition and anthropometric measurements) and (2) the second session was a familiarisation session and further baseline measures. During the familiarisation session, the investigator demonstrated correct form and technique of all eight exercises on the Cybex resistance machines (Cybex VRS, MA, USA). The participants were given the opportunity to perform all the exercises to ensure they were comfortable with the machines and familiarised themselves with the exercises with very light weight. These two sessions were conducted within one-week prior to the first exercise training session.

The independent variable was the mode of exercise; eccentric-only versus concentric-only resistance exercise training as described below. The dependent variables consisted of blood biomarkers (insulin sensitivity, glycemic control and blood lipid profile), body composition, muscle strength, and physical function. Changes in these variables from pre- to post-intervention were compared between groups.

### Exercise intervention

Participants performed supervised resistance exercises (chest press, lateral pulldown, bicep curl, triceps extension, abdominal crunch, leg extension, leg curl and calf raise) on Cybex resistance machines (Cybex VRS, MA, USA) targeting upper- and lower-body muscle groups twice a week on non-consecutive days for 12 weeks, based on the current recommended exercise guidelines for T2D [19]. A periodised and progressive resistance training program was adapted from a previous study [4], with 2–3 sets of 10 repetitions for each exercise for each session (Table 1). Chen et al. [4] found that a similar exercise protocol (3 or 6 sets × 10 repetitions of the knee extensors) performed once a week for 12 weeks was effective for improving insulin sensitivity, glycemic control, and lipid profile in healthy older men, particularly in the eccentric group. The exercise load was based on the one-repetition maximal concentric strength (1-RMcon) measured at baseline for each exercise and gradually increased to 100% of 1-RMcon (Table 1). Due to increases in 1-RMcon throughout the 12 weeks, it should be noted that the 100% load was still submaximal. To minimise delayed onset muscle soreness particularly for the ECC group, the load was set at 10% of 1-RMcon strength in week 1 and progressively increased every 2 weeks up to 100% of 1-RMcon strength in week 12. Participants in the ECC group were instructed to lower the weight and resist against muscle lengthening actions to elicit eccentric contractions at a guided and slow controlled pace of 5-s. For the CON group, the load increased from 50 to 100% of 1-RMcon strength over 24 sessions. Participants in the CON group were instructed to raise or lift the weights within 1–2 s to a fully extended or flexed position dependent on the exercise. Assistance was provided to lower or raise the weights to enable the participant to perform eccentric-only or concentric-only contractions. The rest period between repetitions was approximately 3-s, and 60-s between sets for both ECC and CON. The Borg’s CR-10 rating of perceived exertion (RPE) scale was used to assess the participant’s effort immediately after each exercise and session. Muscle soreness was assessed 48–72 h after each session using a 10-cm visual analog scale (VAS) anchored “0: no pain” and “10: maximal pain imaginable”.

**Table 1:** Resistance exercise training load and volume for the ECC group and CON group (CON) for 24 training sessions over 12 weeks

Week	Session	Eccentric training		Concentric training	
		Load	Volume	Load	Volume
1st	1	10%	2 × 10	50%	2 × 10
	2				
2nd	3	20%	3 × 10	60%	3 × 10
	4				
3rd	5	40%	2 × 10	70%	2 × 10
	6				
4th	7	40%	3 × 10	70%	3 × 10
	8				
5th	9	60%	2 × 10	80%	2 × 10
	10				
6th	11	60%	3 × 10	80%	3 × 10
	12				
7th	13	75%	2 × 10	90%	2 × 10
	14				
8th	15	75%	3 × 10	90%	3 × 10
	16				
9th	17	90%	2 × 10	95%	2 × 10
	18				
10th	19	90%	3 × 10	95%	3 × 10
	20				
11th	21	100%	2 × 10	100%	2 × 10
	22				
12th	23	100%	3 × 10	100%	3 × 10
	24				
TWL (kg)		112,955.0 ± 45,649.0		148,832.0 ± 43,131.0	

Exercise intensity and training volume (number of sets × number of contractions) were progressively increased over the 24 sessions. The total weight lifted (TWL) over 24 training sessions for all eight exercises (mean ± SD) is shown in the last row

### Primary outcome measure

#### Blood biomarkers

Blood samples were obtained in the morning after a 10-h overnight fast at baseline, mid-intervention and post-intervention by a qualified phlebotomist. Venous blood samples were collected from a superficial vein on the radial aspect of the arm by a standard venipuncture, and drawn into the following vacutainer tubes: a 4-ml K2EDTA, a 6-ml fluoride oxalate and a 9-ml serum separator (SST) tube (Vacuette Tube, Greiner Bio-One, Austria). The SST sample was left to clot for 10 min at room temperature and all samples were centrifuged at 3000 rpm for 10 min at 4 °C (Thermo Scientific Heraeus Multifuge 3S-R Centrifuge, Lagenselbold, Germany), aliquoted to several sampling tubes and stored in a − 80 °C alarmed controlled freezer (Thermo Scientific Forma 88000 Series Upright Freezer, Lagenselbold, Germany). The samples were analysed for plasma glucose, serum insulin, HbA1c, triglycerides, total cholesterol, high-density lipoprotein (HDL) and low-density lipoprotein (LDL). Insulin resistance was calculated using the updated homeostasis model assessment based on the following formula: [HOMA2-IR = fasting plasma glucose (mmol/L) × fasting serum insulin (uU/ml)/22.5].

### Secondary outcome measures

#### Body composition and anthropometry measurements

Body composition was measured using a dual energy X-ray absorptiometry imaging scanner (Horizon DXA System, Hologic Inc., MA, USA) by a trained technician. Each participant was instructed to lie supine and still on a scanning bed with their palms faced down, feet position shoulder width apart and toes angled towards each other. The scanning process lasted approximately 3–4 min. On completion, the scans were analysed using the Hologic QDR Software for Windows (Hologic, Bedford, MA, USA), which integrates whole-body measurement and standard body regions including the upper- and lower-limb, and trunk, delineated by specific anatomical landmarks. Whole-body fat mass (FM) and lean mass (LM) in kg, body fat percent (%), and regional tissue composition were determined by manipulation of the segmental lines according to specific anatomical landmarks [44] with appendicular skeletal muscle mass (ASM) calculated from the sum of upper- and lower-limb LM [16].

Height and body mass were measured using a wall-mounted stadiometer (Livingstone International Healthcare Pty Ltd, Australia) and a calibrated electronic weight scale (Model #22089, SECA, Germany) to an accuracy of 0.5 cm and 0.1 kg, respectively. BMI was calculated from the mass divided by the height in meters squared (kg/m^2^). The waist to hip ratio (WHR) was calculated from the measurements of waist circumference (between the lowest rib and iliac crest) and hip circumference (around the largest protrusion of the buttocks) using a flexible tape measure.

#### Muscle strength

Dynamic maximal muscle strength for all eight exercises was assessed at baseline, 6-week and post-intervention using 1-RMcon, which was the maximal weight that could be lifted once with correct technique. The protocol for assessing each participant’s 1-RMcon was as follows; (1) each participant performed one set of six repetitions at approximately 60% of the participant’s perceived maximal strength, (2) after two minutes of rest, the participant performed three repetitions at approximately 80% of the participant’s perceived maximal strength, and (3) the load was gradually increased with 2 min rest between attempts until the participant failed to complete a full repetition with correct technique [43]. RPE was recorded after each attempt to ensure that the participant was able to tolerate the load and intensity. The load for 1-RMcon was recorded as the last successful attempt for each exercise. This measurement was also used to determine the individual exercise load for each participant.

#### Physical function and balance

A battery of physical function tests including a six-minute walk test (6MWT), 5-repetition chair rise test (CR), and 3-m timed up-and-go test (TUG) were performed at baseline and post-intervention. Three trials were performed for CR and TUG with one-minute rest between trials but the 6MWT was performed once.

The 6MWT is one of the most valid and common measures of physical functional capacity [14]. For the 6MWT, a 20-m track was set up with cones at each end of an unobstructed corridor. Each participant was instructed to walk back and forth as quickly as possible for six minutes. A digital stopwatch (A601X Accusplit Pro Survivor Stopwatch, CA, USA) was used to monitor the time, with the investigator notifying the participant of the amount of time left at each minute. At the end of six minutes, the participant was requested to stop walking, and the distance was measured.

The CR test is widely used to measure lower limb strength and endurance [1]. For the CR test, a chair was positioned up against the wall and the participant was instructed to be seated with their back against the back rest, arms folded across the chest and feet positioned shoulder width apart on the floor. The participant was instructed to rise to a full standing position and sit back down with their back touching the back rest, repeating five times as quickly as possible. The fastest performance of the three trials was recorded for further analysis.

The TUG test is used to assess mobility and balance [15]. In the TUG test, a chair was placed up against the wall and the distance of three meters from the chair was marked with a cone. Each participant was instructed to be seated in a similar position as the CR test. The participant was instructed to rise from the chair (without using the hands for support), walk as fast as possible, turn around the cone positioned at the end of the route, return and sit back down on the chair with their back against the back rest. The fastest time of the three trials was recorded for further analysis.

A sensory organisation test (SOT) was performed on the Neurocom Smart Balancemaster (NeuroCom Balance Manager, WA, USA) to assess the participant’s static and dynamic balance using various conditions including eyes open and eyes closed. The participant was required to stand as still as possible with both hands beside their body during each condition while the equilibrium score was quantified. The total score was reported between 0 to 100. A score close to 100 indicates good stability and minimum sway whereas swaying to the limits of stability received a very low score. A score of 0 was automatically assigned to all falls or stopped trials.

### Statistical analysis

The sample size was estimated by a-priori power analysis using G*Power (http://www.gpower.hhu.de/). Based on the effect size of 0.25 for a possible difference in the changes in fasting insulin and glucose concentrations in the blood between the two exercise groups from a previous study [4], with the α-level of 0.05 and a statistical power (1 − β) of 0.80, it was found that at least 16 participants were required for this study. All statistical analyses were performed using the IBM SPSS v27.0 statistical package (SPSS Inc., Chicago, IL, USA). Data was assessed by a Shapiro–Wilk test for normality and a Levene test for the homogeneity of variance assumption. Baseline values of each variable were compared between ECC and CON groups by an independent samples t-test. Changes in each dependent variable over time were compared between the ECC and CON groups by a repeated measures mixed-design two-way ANOVA. When a significant interaction (group × time) effect was found, a Tukey’s post-hoc test was performed to identify and compare the differences between groups for each time point. The changes in each dependent variable from pre- to post-training were also compared between groups by independent t-test. Effect sizes (ES) was calculated using Cohen’s *d* represented as ES = (Mean_Post_ − Mean_Pre_)/SD_Pre_ and was considered as small (*d* > 0.2), medium (*d* > 0.5), and large effect (*d* > 0.8) [6]. All statistical results are shown in mean ± standard deviation (SD) unless otherwise stated.

## Results

The participants completed all 24 sessions of the exercise intervention as planned. Both groups completed the same number of total muscle contractions (600 contractions) for each of the eight exercises over the 12-week period. The total weight lifted was 112,955.0 ± 45,649.0 kg for the ECC group and 148,832.0 ± 43,131.0 kg for the CON group, without a significant difference between groups (Table 1).

**Fig. 2:**
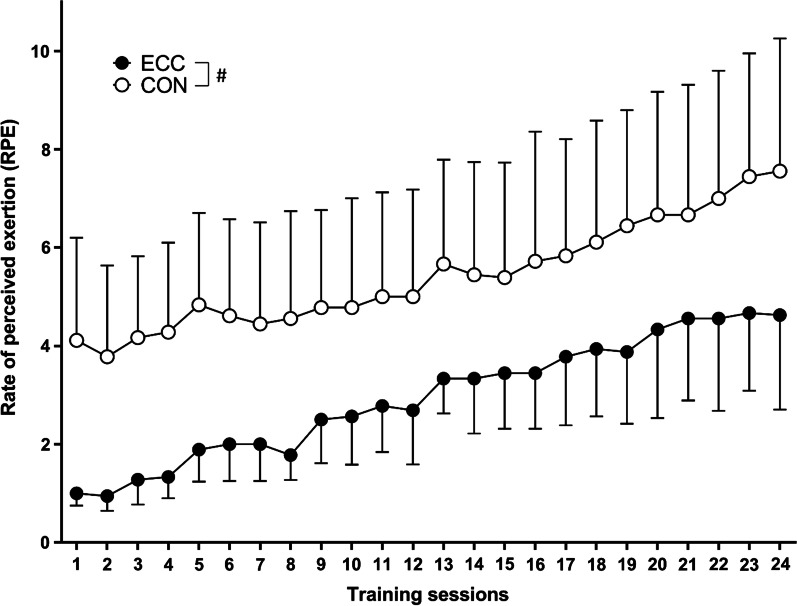
Changes (mean ± SD) in the average sessional RPE between the ECC and CON groups recorded after each exercise training session for 12 weeks (24 sessions). Significant (*p* < 0.05) difference between ECC and CON for all sessions

Sessional RPE increased over 24 sessions as the exercise load gradually increased for both groups (Fig. 2). On average, the CON group elicited a higher RPE (5.4 ± 1.1) than the ECC group (2.9 ± 1.2). At week 12 of the intervention, both groups performed 3 sets of 10 repetitions at a load of 100% 1-RMcon. The average sessional RPE was 4.1 ± 2.1 for the ECC group, and 7.6 ± 2.7 for the CON group, with a significant difference between groups.

Minimal muscle soreness (VAS < 20 mm) in both groups were observed 24–48-h after several exercise sessions and even after the higher-intensity sessions. No significant difference was found between groups.

### Blood biomarkers

As shown in Table 2, no significant difference between groups was found for any of the blood markers at baseline. Significant changes (*p* < 0.05) including increases in fasting serum insulin (2.7 ± 1.3 mU/L) and HOMA2-IR (0.4 ± 0.1), and decreases in HbA1c (− 0.4 ± 0.1%) were evident after 6 weeks of training in the ECC group only. HbA1c significantly decreased (*p* < 0.05) after 12 weeks of concentric training (− 0.3 ± 0.0%) but not in the ECC group (− 0.1 ± 0.1%). No significant changes were found in lipid profile for both groups from pre- to post-intervention.

**Table 2:** Changes (mean ± SD) in blood diabetes markers (fasting plasma glucose, fasting serum insulin, HOMA-2 IR, HbA1c) and blood lipid profile (triglycerides, total cholesterol, HDL, LDL) before (Pre), after 12 (Mid) and 24 sessions (Post) of resistance training for the ECC and CON groups.

Variables	Eccentric (*n* = 9)					Concentric (*n* = 9)					Effect size
	Pre	Mid	Post	Pre versus Post Mean difference [95% CI]	Pre versus Post *p*	Pre	Mid	Post	Pre versus Post Mean difference [95% CI]	Pre versus Post *p*	ECC versus CON
*Blood diabetes profile*											
Glucose (mmol/L)	7.9 ± 1.9	7.9 ± 1.6	8.1 ± 1.5	− 0.2 [− 1.5 to 1.2]	0.829	6.8 ± 1.7	6.9 ± 1.1	6.7 ± 1.2	0.1 [− 0.8 to 0.9]	0.906	0.163
Insulin (mU/L)	9.1 ± 3.2	11.8 ± 4.5*	10.4 ± 4.5	− 1.3 [− 3.9 to 1.2]	0.267	8.7 ± 4.1	10.5 ± 6.2	10.8 ± 6.3	− 2.1 [− 4.6 to 0.4]	0.092	0.179
HOMA-2 IR	1.3 ± 0.5	1.7 ± 0.6*	1.5 ± 0.6	− 0.2 [− 0.5 to 0.2]	0.258	1.2 ± 0.5	1.4 ± 0.8	1.5 ± 0.9	− 0.3 [− 0.7 to 0.1]	0.112	0.194
HbA1c (%)	7.2 ± 0.7	6.8 ± 0.8*	7.1 ± 0.8	0.1 [− 0.2 to 0.4]	0.502	6.6 ± 0.5	6.2 ± 0.7	6.3 ± 0.6*	0.3 [0.1 to 0.6]	0.024	0.668
*Blood lipid profile*											
Triglycerides (mmol/L)	1.4 ± 0.5	1.6 ± 0.8	1.9 ± 1.5	− 0.5 [− 1.4 to 0.5]	0.280	1.4 ± 0.5	1.3 ± 0.5	1.3 ± 0.5	0.1 [− 0.2 to 0.3]	0.617	0.544
Cholesterol (mmol/L)	5.2 ± 0.7	5.1 ± 0.7	5.1 ± 0.9	0.1 [− 0.2 to 0.5]	0.386	4.5 ± 1.3	4.4 ± 1.4	4.4 ± 1.4	0.1 [− 0.3 to 0.4]	0.580	0.032
HDL (mmol/L)	1.3 ± 0.2	1.3 ± 0.2	1.3 ± 0.2	0.0 [− 0.1 to 0.1]	0.665	1.5 ± 0.6	1.4 ± 0.5	1.4 ± 0.4	− 0.1 [0.0 to 0.2]	0.137	0.485
LDL (mmol/L)	3.2 ± 0.7	3.0 ± 0.7	3.0 ± 0.7	0.2 [− 0.1 to 0.6]	0.116	2.3 ± 0.9	2.4 ± 1.1	2.4 ± 1.2	− 0.1 [− 0.4 to 0.3]	0.822	0.453

The results of comparison between Pre and Post by t-test with p values shown in the column of Pre versus Post for each group

*Indicates a significant (*p* < 0.05) difference from the Pre value. Effect size for the difference between ECC and CON groups is shown in the right column

### Body composition and anthropometry measurements

Significant reductions in FM (ECC − 2.1 ± 1.3 kg, *p* = 0.025; CON − 2.2 ± 1.2 kg, *p* = 0.005) and body fat % (ECC − 2.0 ± 0.6%, *p* = 0.017; CON − 2.3 ± 0.1%, *p* = 0.001) were found from pre- to post-intervention; however, no significant differences were evident between groups (Table 3). Total LM increased significantly after training for both ECC (1.8 ± 0.8 kg, *p* = 0.034) and CON (2.0 ± 0.3 kg, *p* = 0.015) groups.

**Table 3:** Changes (mean ± SD) in anthropometry measures, fat mass and lean mass from baseline (Pre) to post-intervention (Post) for ECC and CON groups

Variables	Eccentric (*n* = 9)				Concentric (*n* = 9)				Effect size
	Pre	Post	Pre versus Post Mean difference [95% CI]	Pre versus post *p*	Pre	Post	Pre versus Post Mean difference [95% CI]	Pre versus Post *p*	ECC versus CON
*Anthropometry measures*									
Body mass (kg)	90.4 ± 16.3	90.5 ± 15.5	− 0.1 [− 1.6 to 1.4]	0.934	89.5 ± 15.3	89.4 ± 14.2	0.1 [− 1.3 to 1.5]	0.861	0.070
Body mass index (kg/m^2^)	30.3 ± 5.2	30.2 ± 4.7	0.1 [− 0.4 to 0.5]	0.786	29.4 ± 3.8	29.5 ± 3.3	− 0.1 [− 0.7 to 0.4]	0.568	0.270
Waist (cm)	104.4 ± 14.3	99.2 ± 13.3*	5.2 [2.9 to 7.5]	0.001	99.28 ± 12.07	95.0 ± 11.31*	4.2 [1.5 to 7.1]	0.008	0.198
Hip (cm)	104.5 ± 9.42	103.17 ± 8.40	1.3 [− 0.5 to 3.2]	0.132	105.17 ± 11.3	100.4 ± 10.69*	4.7 [2.0 to 7.4]	0.004	1.174
Waist-to-hip ratio	0.997 ± 0.079	0.960 ± 0.077*	0.0 [0.0 to 0.0]	0.036	0.945 ± 0.072	0.948 ± 0.078	0.0 [0.0 to 0.0]	0.862	0.840
*Fat mass (kg)*									
Total body	33.6 ± 10.6	31.5 ± 9.3*	2.0 [0.3 to 3.7]	0.025	30.7 ± 7.6	28.5 ± 6.4*	2.2 [0.9 to 3.5]	0.005	0.545
Upper-limb	3.7 ± 1.2	3.5 ± 1.1	0.2 [0.0 to 0.4]	0.090	3.5 ± 0.8	3.1 ± 0.7*	0.4 [0.2 to 0.6]	0.003	0.982
Lower-limb	10.5 ± 3.2	9.9 ± 2.9*	0.6 [0.1 to 1.1]	0.024	9.5 ± 2.8	9.1 ± 2.4	0.4 [− 0.1 to 0.8]	0.115	0.281
Trunk	18.0 ± 6.9	16.8 ± 6.1	− 0.7 [− 1.4 to 0.0]	0.092	16.4 ± 4.7	15.1 ± 3.6*	1.0 [0.2 to 2.6]	0.029	0.259
Body fat (%)	36.6 ± 8.6	34.6 ± 8.0*	2.0 [0.5 to 3.5]	0.017	34.1 ± 6.5	31.8 ± 6.4*	2.3 [1.3 to 3.3]	0.001	0.371
*Lean mass (kg)*									
Total body	55.0 ± 10.6	56.8 ± 11.4*	1.8 [3.3 to 0.2]	0.034	57.0 ± 11.0	59.0 ± 11.3*	2.0 [− 3.6 to − 0.5]	0.015	0.148
Upper-limb	5.5 ± 1.5	6.0 ± 1.8*	0.6 [1.7 to 0.5]	0.047	6.2 ± 1.5	6.3 ± 1.6	0.1 [− 2.0 to 0.0]	0.638	0.806
Lower-limb	16.8 ± 3.5	17.4 ± 4.3	0.5 [− 1.0 to 0.0]	0.261	17.7 ± 3.5	18.6 ± 4.1	0.1 [− 0.5 to 0.4]	0.070	0.204
Trunk	29.6 ± 5.7	30.3 ± 5.5	0.7 [− 1.4 to 0.0]	0.063	30.0 ± 6.2	31.0 ± 5.5*	1.0 [− 2.0 to 0.0]	0.037	0.395
Appendicular skeletal mass	22.3 ± 4.9	23.5 ± 6.0	1.1 [− 2.6 to 0.4]	0.119	23.8 ± 4.9	24.9 ± 5.6	1.0 [− 2.2 to 0.0]	0.064	0.107

The results of comparison between Pre and Post by t-test with *p* values shown in the column of Pre versus Post for each group

*Indicates a significant (*p* < 0.05) difference from the Pre value. Effect size for the difference between ECC and CON groups is shown in the right column

As shown in Table 3, none of the anthropometry measures were significantly different between groups at baseline. Significant decreases (*p* < 0.05) in waist circumference were found for both ECC (− 5.2 ± 1.0 cm) and CON (− 4.3 ± 0.8 cm) groups from pre- to post-intervention. A significant decrease in hip circumference was found for the CON group only (− 4.8 ± 0.6 cm), and a significant decrease in the waist-to-hip ratio was evident for the ECC group only (− 0.038 ± 0.002) from pre- to post-intervention.

### Muscle strength

**Fig. 3:**
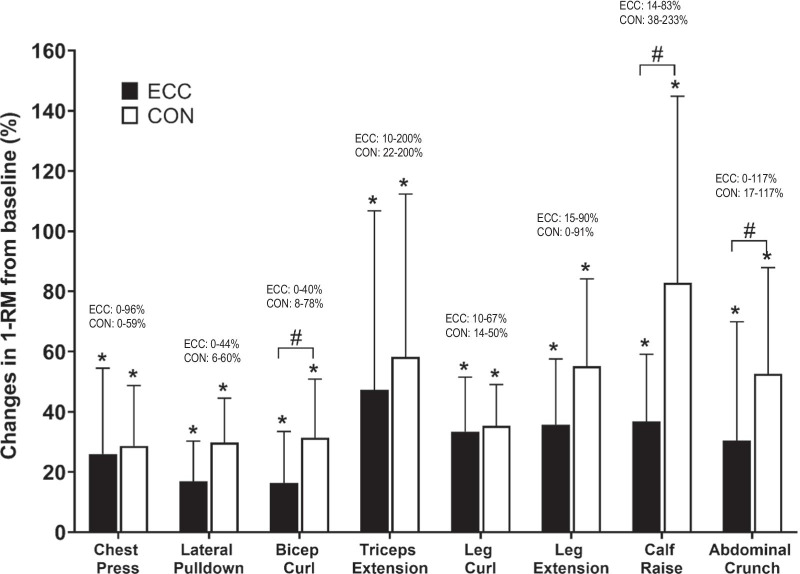
Changes (mean ± SD) in muscle strength assessed by concentric one-repetition maximum (1-RMcon) from baseline to post-intervention for each of the eight exercises for the eccentric resistance training group (ECC) and concentric resistance training group (CON) and individual range of % change. *Indicates significant changes from baseline (*p* < 0.05). ^#^Indicates significant difference between groups (*p* < 0.05)

No significant difference between groups was found for any of the baseline muscle strength measures. Significant increases were found in upper- and lower-body strength (*p* < 0.05) for all eight exercises in both groups (Fig. 3). Upper-body strength increased between 12–33% in the ECC group and 27–43% in the CON group, while lower-body strength increased between 32–37% in the ECC group and 34–68% in the CON group. Significant differences were found between the ECC and CON groups for bicep curl, calf raise and abdominal crunch exercises (*p* < 0.05) with the CON group showing greater increases.

### Physical function and balance

The baseline values were not significantly different between groups for physical function and balance measures (Table 4). After 12 weeks of training, both groups showed improvements (*p* < 0.05) for 6MWT distance (ECC 56.8 ± 2.2 m, CON 63.4 ± 12.0 m) and CR time (ECC − 1.8 ± 1.3 s, CON − 2.3 ± 1.6 s) without significant differences between groups. Significant improvements for TUG were found only for the ECC group (− 0.8 ± 0.2 s). No significant changes before and after the 12-week training intervention were found for both groups in the balance tests including the overall score (Table 4).

**Table 4:** Changes (mean ± SD) in physical function assessed by six-minute walk test, five repetition chair rise time and 3-m timed up-and-go, and balance sensory measures for the total score, eyes closed and sway vision from baseline (Pre) to post-intervention (Post) for ECC and CON groups

Variables	Eccentric (*n* = 9)				Concentric (*n* = 9)				Effect size
	Pre	Post	Pre versus post mean difference [95% CI]	Pre versus post *p*	Pre	Post	Pre versus post Mean difference [95% CI]	Pre versus post *p*	ECC versus CON
*Physical function*									
6-min walk test (m)	463.9 ± 94.1	520.7 ± 91.9*	− 46.8 [− 98.1 to 4.6]	0.018	507.9 ± 79.9	571.3 ± 67.9*	− 59.7 [− 95.9 to − 23.5]	0.005	0.083
5-rep chair rise (s)	13.4 ± 5.1	11.6 ± 3.8*	1.8 [0.4 to 3.1]	0.015	11.5 ± 3.0	9.2 ± 1.4*	2.3 [0.4 to 4.3]	0.023	0.463
3-m timed-up and go (s)	7.1 ± 2.2	6.3 ± 1.9*	0.6 [0.0 to 1.2]	0.005	5.8 ± 0.8	5.3 ± 0.5	0.5 [0.0 to 1.0]	0.055	0.014
*Balance sensory measures*									
Total composite score	71.3 ± 11.1	76.3 ± 6.3	− 5.0 [− 11.0 to 1.0]	0.089	74.3 ± 6.6	77.6 ± 5.5	− 3.2 [− 8.0 to 1.6]	0.162	0.325
Eyes closed	46.4 ± 20.7	63.2 ± 10.1	− 16.9 [− 35.1 to 1.3]	0.065	57.9 ± 11.1	62.6 ± 9.6	− 4.7 [− 16.0 to 6.6]	0.369	0.481
Sway vision	50.3 ± 27.7	58.1 ± 18.2	− 7.9 [− 18.9 to 3.1]	0.137	54.8 ± 13.6	61.8 ± 10.2	− 7.0 [− 18.6 to 4.6]	0.201	0.493

*Indicates a significant (*p* < 0.05) difference from the Pre value. Effect size for the difference between ECC and CON groups is shown in the right column

## Discussion

To the best of our knowledge, this was the first study to compare the effects of progressive eccentric-only versus concentric-only resistance exercise training on biochemical and physical outcome measures in patients with T2D. No significant changes were found in blood diabetes and lipid profile markers at the end of the intervention for both training groups (Table 2). However, significant improvements in body composition (Table 3), muscle strength (Fig. 3) and physical function (Table 4) were observed after 12 weeks of both eccentric-only and concentric-only resistance training. Thus, the hypothesis that eccentric-only resistance training would improve all outcome measures better than concentric-only resistance training was not supported by the results. However, it should be noted that the average RPE during training was less for ECC (2.9 ± 1.2) than CON (5.4 ± 1.1) as shown in Fig. 2. It is important to note that the effects of resistance training on the outcome measures were induced by less strenuous exercise sessions in the eccentric-only than concentric-only training.

As shown in Fig. 2, the average RPE in the training sessions was “hard” for the CON group but “somewhat moderate” for the ECC group. The ECC group reported an average RPE of 4.1 ± 2.1 (moderate) while the CON group’s RPE was 7.6 ± 2.7 (very hard) after performing 3 sets of 10 repetitions at 100% of baseline 1-RMcon during sessions 21–24. This was in line with the findings of previous studies showing that eccentric exercise was easier to perform than concentric exercise for the same absolute workload [11, 26, 37, 38]. Importantly, minimal or no muscle soreness was reported after any exercise session in both groups even at high-intensity sessions. This was also evident in a previous study [4] and is likely due to the protective effect conferred by progressive overload which commenced from a very low intensity. This is important as muscle soreness after eccentric exercise can be a factor that discourages people from continuing regular exercise [18]. Using the progressive training protocol, the participants appeared to be able to adhere to the exercise training program over 12 weeks and achieved significant gains in muscle strength.

As shown in Table 2, the present study did not find any significant changes in blood diabetes markers following eccentric resistance training. Chen et al. [4] showed significant improvements in insulin sensitivity including reductions in fasting glucose, insulin, HOMA and HbA1c following 12 weeks of eccentric resistance training of the knee extensors in healthy older men. In the present study, the ECC group showed improvements in HbA1c after 6 weeks but not after 12 weeks of training. This difference could be attributed to prescribed medications consumed, which was recorded but not controlled in the present study. The effects of ECC resistance training on individuals with prescribed T2D medications (including the commonly prescribed Metformin) remains to be determined [34]. Muscle strength gains, increases in insulin sensitivity and improvements in glycemic control are considered normal adaptations to exercise training; however, some studies have found that Metformin inhibits muscle hypertrophy and blunts the effect of muscle mass gains in response to progressive resistance training in older adults [2, 28, 45]. The combination of Metformin and exercise may be less effective in reducing glycemic response [3] and may in fact attenuate the well-documented benefits of exercise alone [42]. In fact, Sharoff et al. [42] reported that exercise alone increased insulin sensitivity; however, a combination of exercise and Metformin did not enhance insulin sensitivity and showed an increase in glucose production due to less AMP-activated protein kinase (AMPK) activation. It is also possible that the effects of the exercise training on insulin sensitivity markers were masked by the effects of Metformin. Eighty percent of the participants in this study took Metformin as a prescribed diabetes medication which may explain the lack of significant changes in insulin sensitivity and glycemic control.

Paschalis et al. [33] found favorable changes in lipid profile including decreases in total cholesterol levels (− 8.8%), triglycerides (− 12.8%) and LDL (− 16.4%) after performing eccentric resistance training once a week for 8 weeks in a group of healthy women. Chen et al. [4] also showed significant changes in blood lipid markers (TC − 8%, TG − 16%, LDL − 8%) after 12 sessions of knee extensor eccentric training over 12 weeks. However, the present study did not find any significant changes in blood lipid profile following ECC or CON training. The average fasting cholesterol levels were 5.6 mmol/L and 4.5 mmol/L respectively for the ECC and CON groups. Critically, participants in this study had near-normal levels of lipid profile parameters observed at baseline; thus, the ceiling effect might explain no positive changes in blood lipid profile.

Both the ECC and CON groups showed a significant increase of approximately 3% in whole-body LM after 12 weeks of training (Table 3). Marcus et al. [30] reported an increase of up to 10.5% in thigh lean mass and decrease of − 1.2 cm around the thigh-intramuscular fat cross-sectional area using magnetic resonance imaging (MRI) scans after 16 weeks of high-force eccentric resistance exercises in combination with aerobic exercise in adults with T2D. Importantly, segmental LM significantly increased in the upper limb (9.1%), lower limb (3.6%) and abdominal (2.4%) regions for the ECC group only (Table 3). Simultaneous gains in LM and decrease in body fat % are important in attenuating muscle wastage in the elderly [31]. In the present study, both groups demonstrated significant decreases in body fat % including reductions in trunk fat, waist circumference, and total FM. We found a significant decrease of 2.1 kg in total body mass in the ECC group, with majority of the fat loss around the abdominal region. Julian et al. [22] observed decreases in whole-body (− 10%) and leg FM (− 6.5%) following 12 weeks of eccentric cycling training in obese adolescents, and stated that these improvements could be due to large increases in post-training resting energy expenditure after eccentric exercise. Gluchowski et al. [12] also found that eccentric exercise prescription was beneficial in improving body composition and could potentially be an important stimulus in preventing sarcopenia, osteoporosis, and obesity. However, the specific differences between eccentric and concentric muscle contractions remain unclear and require further investigation.

Eccentric exercise training increases muscle strength with lesser perceived effort when compared to concentric exercise [21]. Results from a meta-analysis showed that high-intensity eccentric resistance exercise was superior to concentric resistance exercise in stimulating muscle strength increases. This is possibly due to the higher force developed during eccentric contractions contributed through neurological, physiological and architectural changes [39]. Older adults seem to preserve greater residual force enhancement after eccentric contractions and can produce eccentric strength more efficiently than isometric and concentric strength, which may be a contributing factor to improvements in muscle strength [36]. Chen et al. [4] compared the effects of eccentric and concentric knee extension exercise performed once a week for 12 weeks and found greater increase in 1-RMcon strength in the eccentric group (49%) than the concentric group (35%). In contrast, the present study found that 1-RMcon knee extension strength had smaller increases for the ECC (36%) than CON training (55%) over 12 weeks (24 sessions). Due to the large variability among the participants for changes in the 1-RMcon strength, no significant difference between ECC and CON was detected for knee extension strength. Although the CON group commenced exercise load at 50% of 1-RMcon in comparison to the ECC group at 10%, the average total weight lifted over 24 sessions was not significantly different between CON and ECC groups. When comparing ECC and CON for each exercise, significant differences in the total weight lifted were found for the triceps extension only (CON > ECC). It is important to note that the increases in 1-RMcon strength was larger in the CON group for bicep curl, calf raise and abdominal crunch exercises than the ECC group. The magnitude of changes in 1-RMcon strength varied among the exercises such that the largest increase was found for calf raise (ECC 37%, CON 68%) and the smallest increase was found for bicep curl (ECC 11%, CON 27%). It should also be noted that the 1-RM measurement was performed concentrically; thus, participants in the CON group might have advantages due to the specificity principle. Future studies could potentially include other strength measures such as maximal voluntary isometric and/or isokinetic contraction strength to measure force and velocity.

Following the 12-week intervention, participants exhibited improvements in physical function demonstrated by faster times recorded for the CR (ECC 13.4%, CON 20.0%) and TUG tests (ECC 11.3%, CON 8.6%), and increased distance for the 6MWT (ECC 12.2%, CON 12.5%) when compared with the baseline values (Table 2). Our results were consistent with the findings of previous studies [9, 37] reporting that eccentric resistance exercise was efficient and effective in improving functional capacity in older adults. Raj et al. [37] reported similar improvements in functional performance after 16-weeks of eccentrically-biased resistance training (6MWT 7%, TUG 3%) and conventional resistance training (6MWT 5%, TUG 5%) consisting of leg press, toe press, bench press and lateral pulldown. Dias et al. [9] reported significant improvements in 6MWT (12%), CR (15%) and TUG (16%) after 6 weeks of eccentric-focused resistance training consisting of leg press, seated row, knee extension and bench press, although the leg press 1-RMcon did not change significantly throughout the study. It appears that the hamstring muscle is a vital muscle in walking ability as it controls and stabilises the hip movement. The degree of hip and knee flexion appears to increase to maintain the body’s center of gravity as walking speed increases [29]. This is important in maintaining physical functional performance and balance in adults with chronic disease. The balance ability assessed by SOT did not show significant changes after ECC or CON training (Table 2). Previous studies [4, 23] showed greater improvements in balance after eccentric than concentric resistance training using different tests (Berg balance test, static balance test with eyes open and closed). It is important to note that all the participants achieved scores above 80% during their baseline testing and this ceiling effect may be the reason for no further improvement found in this study [40].

There were several limitations in the present study, which should be considered for future studies. Firstly, the sample size was small as many participants recruited for the study did not want to commit to three months of continuous training. Secondly, no control group without any exercise intervention was included in the present study. Although, it is reasonable to assume that no changes in the outcome measures would have been found for the control group. Thirdly, the participants were asked to record a 3-day food diary; however, they were not required to restrict their caloric intake or other physical activities during the intervention. Even though the participants were recommended not to change their eating, drinking or physical activity habits, additional lifestyle and behavioral changes due to the positive effects of participating in an exercise intervention could not be entirely ruled out. Fourthly, the exercise protocol of the present study required the investigators to physically assist and lift heavy loads to eliminate the eccentric or concentric phase. The practical applications of performing eccentric only resistance exercises must be considered, as specific eccentric-only equipment is costly and may not be readily available in a clinical setting. It should also be noted that the exercise intensity and the volume were not matched between the eccentric and concentric groups. It is possible to increase the load for the eccentric-only exercises, since maximal voluntary contraction strength is greater for eccentric than concentric contractions. Future studies should investigate if eccentric and concentric resistance training of the same absolute workload, volume and/or RPE at an increased frequency can elicit greater improvements in blood markers for the eccentric-only than concentric-only training.

## Conclusion

In summary, no significant changes in blood biomarkers were found after the 12-week intervention and no significant differences were found between the two groups; thus, our hypotheses were not supported. Nevertheless, there were some noteworthy findings in the present study. All participants were able to tolerate and perform the exercises with minimal muscle soreness and no injury, contraindications, or adverse events. Our findings demonstrated that commencing eccentric resistance training at a lower intensity with gradual progression can elicit significant exercise training adaptations and positive outcomes in improving body composition, muscle strength and physical function for adults with T2D. This is important to prevent physical decline and sarcopenia and improve overall quality of life particularly for older adults who have limited functional capacity and clinical health conditions.

## Data Availability

The datasets generated and analysed that support the findings of this study are available on reasonable request from the corresponding author [CK]. The data is not publicly available due to them containing information that could compromise research participant privacy/consent.

## Abbreviations

1-RM: One-repetition maximum
6MWT: Six-minute walk test
ANOVA: Analysis of variance
ASM: Appendicular skeletal muscle mass
BMI: Body mass index
CON: Concentric resistance training
CR: Chair rise
ECC: Eccentric resistance training
ES: Effect size
HbA1c: Glycosylated hemoglobin
HDL: High-density lipoprotein levels
HOMA2-IR: Homeostatic Model Assessment for Insulin resistance
LDL: Low-density lipoprotein levels
RPE: Rate of perceived exertion
SD: Standard deviation
SOT: Sensory organization test
SST: Serum separator tube
T2D: Type 2 diabetes
TUG: Timed up-and-go
